# Prognostic Role of Pyruvate Kinase M2 in High-Grade Gliomas: A Quantitative Immunohistochemistry Study

**DOI:** 10.7759/cureus.78035

**Published:** 2025-01-26

**Authors:** Corina Tamas, Alina R Cehan, Attila Kövecsi, Flaviu Tamas, Adrian F Balasa

**Affiliations:** 1 Doctoral School of Medicine and Pharmacy, George Emil Palade University of Medicine, Pharmacy, Science, and Technology, Targu Mures, ROU; 2 Department of Pathology, County Emergency Clinical Hospital of Targu Mures, Targu Mures, ROU; 3 Department of Neurosurgery, County Emergency Clinical Hospital of Targu Mures, Targu Mures, ROU

**Keywords:** astrocytoma, atrx, glioblastoma, immunohistochemistry, pkm2

## Abstract

Background: Glioblastoma (GBM) and grade 4 astrocytoma (ASTROG4) are aggressive primary brain tumors characterized by rapid growth, invasiveness, and poor prognosis, differentiated by the presence or absence of isocitrate dehydrogenase (IDH) mutation according to the World Health Organization (WHO) 2021 classification. Essential molecular markers, in addition to IDH mutations, include alpha-thalassemia/mental retardation syndrome X-linked (ATRX) loss and p53 expression, which significantly influence their classification and prognosis. Pyruvate kinase M2 (PKM2), a critical enzyme in tumor metabolism, has been implicated in glioma progression, but its prognostic significance remains unclear.

Methods: This prospective study aimed to quantitatively measure PKM2 immunohistochemistry (IHC) expression in GBM (IDH wildtype) versus ASTROG4 (IDH R132H mutant), to assess the correlation between PKM2 expression and prognosis in these two patient groups, and to investigate the prognostic significance of ATRX and p53 expression in relation to PKM2 levels. A total of 67 patients with high-grade gliomas (43 GBM, 24 ASTROG4) were analyzed using IHC for IDH1, ATRX, p53, and PKM2. PKM2 expression was quantified using 3DHISTECH (Budapest, Hungary) image analysis software, and correlations with clinical parameters, survival, and other molecular markers were evaluated. Kaplan-Meier survival analysis and Cox regression models assessed the impact of PKM2 expression and clinical factors on prognosis.

Results: PKM2 expression was observed in both GBM and ASTROG4, with no significant differences in positivity rates. However, high PKM2 intensity scores significantly correlated with increased mortality risk (p=0.041). ATRX-negative tumors showed elevated PKM2 levels, suggesting compensatory metabolic adaptations. ASTROG4 cases had better survival outcomes than GBM. Severe preoperative motor deficits were associated with a threefold increase in mortality risk, highlighting the critical role of clinical factors in determining prognosis.

Conclusions: PKM2 plays an important role in glioma metabolism and can serve as a potential therapeutic target. Its association with ATRX highlights its involvement in tumor progression and genomic instability. Combining molecular markers with clinical parameters can improve prognostic accuracy and inform personalized treatment strategies for astrocytic tumors.

## Introduction

Astrocytic tumors are the most common primary brain tumors in adults, accounting for 86% of the annual cases in Europe, with 63% being high-grade malignancies. A key characteristic of gliomas is the presence or absence of isocitrate dehydrogenase (IDH) enzyme expression. The most common IDH mutation in gliomas is IDH1 R132H, which accounts for up to 91% of IDH mutations. The current WHO 2021 classification of adult gliomas relies heavily on IDH expression. It is well established that the presence of an IDH mutation is associated with slower tumor growth, while IDH wild-type tumors tend to be more aggressive. Thus, according to the WHO 2021 classification, a diffuse glioma exhibiting microvascular proliferation or necrosis, along with an IDH mutation, is classified not as glioblastoma (GBM), but as grade 4 astrocytoma (ASTROG4) [1-6].

In addition to IDH expression, the mutation of tumor protein p53 and the inactivation of the alpha-thalassemia/mental retardation syndrome X-linked (ATRX) pathway are also significant in gliomas. Studies have shown that ATRX expression often accompanies IDH mutation and frequently co-occurs with p53 mutation, suggesting a cooperative pathogenic mechanism between these three proteins essential for oncogenesis. ATRX facilitates the incorporation of the histone variant H3.3 into heterochromatin, leading to telomere length alterations and genomic instability. The TP53 gene plays a critical role in cell regulating cycle arrest, differentiation, apoptosis, senescence, and DNA damage repair. Mutant p53 protein interacts with other oncoproteins, promoting tumorigenesis. P53 is upregulated in many cancers and plays a significant role in the development and progression of astrocytic tumors. The frequency of p53 mutations in astrocytoma is estimated to be between 50-70%. Research continues into the relationship between ATRX and p53 mutations, particularly their prognostic significance and influence on patient survival rates [7-10].

For high-grade glioma, the standard treatment consists of maximal surgical resection followed by radiotherapy and chemotherapy, primarily temozolomide. However, overall one-year survival (OS) is 53.7% for GBM, compared to 98.0%, 92.4%, and 76.3% for grade 2, 3, and 4 astrocytomas, respectively. Thus, there is a pressing need to identify new therapeutic approaches to improve survival rates [11-13].

Tumor metabolism has become a promising target for cancer treatment, including in astrocytic tumors, which exhibit significant metabolic remodeling to support survival, proliferation, and invasion. Understanding the mechanisms and protumorigenic metabolic pathways is essential in identifying relevant therapeutic targets. Since the primary energy source for rapidly growing tissues is glycolytic oxidation of glucose, enzymes involved in glycolysis, such as pyruvate kinases, may serve as important therapeutic targets. During glycolysis, each glucose molecule is converted into two pyruvate molecules and two ATP molecules in the cytoplasm. Pyruvate kinase M2 (PKM2) catalyzes the final irreversible step of glycolysis and serves as a key rate-limiting enzyme in cancer cell metabolism [14-19].

PKM2 overexpression has been observed in various cancers, providing tumor cells with selective growth advantages and contributing to metabolic reprogramming by increasing glucose uptake, inhibiting autophagy, and promoting lactate production. Conversely, downregulation of PKM2 induces cell death and reduces tumor cell proliferation by suppressing aerobic glycolysis. Disrupting glycolysis is considered a promising strategy for eliminating tumor cells, with PKM2 emerging as a potential therapeutic target. In gliomas, increased PKM2 expression has been observed in tumor tissue as well as in the adjacent normal brain tissue, highlighting PKM2’s crucial role in tumorigenesis. While various studies have explored strategies to regulate PKM2 and its therapeutic potential, its precise role in glioma progression and its prognostic significance in astrocytic-origin tumors remain unclear [20-23].

This study aimed to quantitatively measure PKM2 IHC expression in GBM (IDH wildtype) versus ASTROG4 (IDH R132H mutant), to assess the correlation between PKM2 expression and prognosis in these two patient groups, and to investigate the prognostic significance of ATRX and p53 expression in relation to PKM2 levels.

## Materials and methods

Sample source and collection

This prospective, single-center study was conducted in the Department of Neurosurgery and the Department of Pathology at the County Emergency Clinical Hospital in Targu Mures, Romania, between January 2021 and December 2023. The patients were divided into two groups based on IHC classification: subjects presenting the IDH R132H mutation were classified as ASTROG4, while those with IDH wildtype expression were classified as GBM.

During the study period, 144 cases with clinical and imaging suspicions of high-grade gliomas underwent surgical treatment. Of these, 67 cases (43 GBM and 24 ASTROG4) met the inclusion criteria, which were as follows: histopathological confirmation of grade 4 astrocytic-origin glial tumors, newly diagnosed (de novo) tumors, patients who had not received prior oncological treatment, complete tumor resection (defined as the absence of contrast enhancement on postoperative imaging within 72 hours of surgery, MRI T1+c), signed informed consent for study participation, successful histopathological slide analysis using designated software.

The exclusion criteria for the study included the following: patients with incomplete clinical or pathological data, glial tumors of non-astrocytic origin, Grade 2 or 3 astrocytoma, recurrent tumors, subtotal or partial resection, biopsy-only cases, loss of patients to follow-up, refusal to participate in the study, errors in slide readings by the software.

Histology and IHC were both performed according to the WHO 2021 [3] criteria for all included cases (n=67) before the cohorts were divided into GBM (n=43) and ASTROG4 (n=24). This approach ensured that the histological evaluations adhered to standardized guidelines, allowing for reliable comparisons between the groups. The division of the cohorts into these two groups was then based on the IHC results.

Postoperative imaging, performed within the first 72 hours after surgery to evaluate the completeness of tumor resection, followed a standardized MRI protocol. Scans were conducted using a 1.5T Siemens Sonata or Avanto system (Siemens Medical Systems, Erlangen, Germany). The protocol included axial T1-weighted and T2-weighted sequences with a 5-mm section thickness, sagittal fluid-attenuated inversion recovery (FLAIR) images with a 1.3 mm section thickness, and axial diffusion-weighted imaging (DWI) images with a 5 mm section thickness. Diffusion gradients were applied using b-values of 0, 500, and 1000 s/mm², with apparent diffusion coefficient (ADC) maps subsequently calculated. Post-contrast imaging consisted of sagittal 3D T1-weighted gradient-echo (MPRAGE) sequences with a section thickness of 1-1.5 mm.

All participants included in the study provided informed consent. This study was conducted in accordance with the Declaration of Helsinki, and the protocol was approved by the Hospital’s Ethics Committee (No. 30 291 from 08.12.2020). Each patient underwent clinical evaluation at multiple time points: before surgery, 24 hours postoperatively, at discharge, and every three months thereafter. Clinical data were recorded on individual evaluation sheets, which were used throughout the follow-up period. Cognitive function was assessed using the Montreal Cognitive Assessment Test [24], motor function using the Medical Research Council (MRC) scale [25], and aphasia using the Kaplan score [26]. Major motor deficits were defined as an MRC score ≤3.

Immunohistochemistry

The IHC reactions were performed on paraffin-embedded tissue sections with a thickness of 4 μm. For deparaffinization, rehydration, and antigen retrieval, the sections were treated with EnVision FLEX Target Retrieval Solution (pH 9, 20 min, 97°C; Dako, Glostrup, Denmark) using the PTLink platform (Dako). Endogenous peroxidase activity was blocked by a five-minute incubation with EnVision FLEX Peroxidase Blocking Reagent (Dako). Subsequently, the slides were incubated for one hour at 37°C with specific primary antibodies: IDH1-R132H (clone IHC 132, dilution 1:25; Bio SB Inc., Goleta, CA, USA), ATRX (clone BSB 108, dilution 1:50; Bio SB; p53: clone DO7, dilution 1:800; Bio SB), PKM2 (polyclonal antibody, unconjugated, RRID: AB_2546815). The EnVision Flex/horseradish peroxidase (HRP) secondary system (Dako, 30 min) was used for signal amplification, and the chromogen 3,3′-diaminobenzidine (DAB) was used for primary antibody detection. The slides were then counterstained with hematoxylin. IHC reactions for each antibody were performed separately for each patient on a distinct slide.

**Figure 1:**
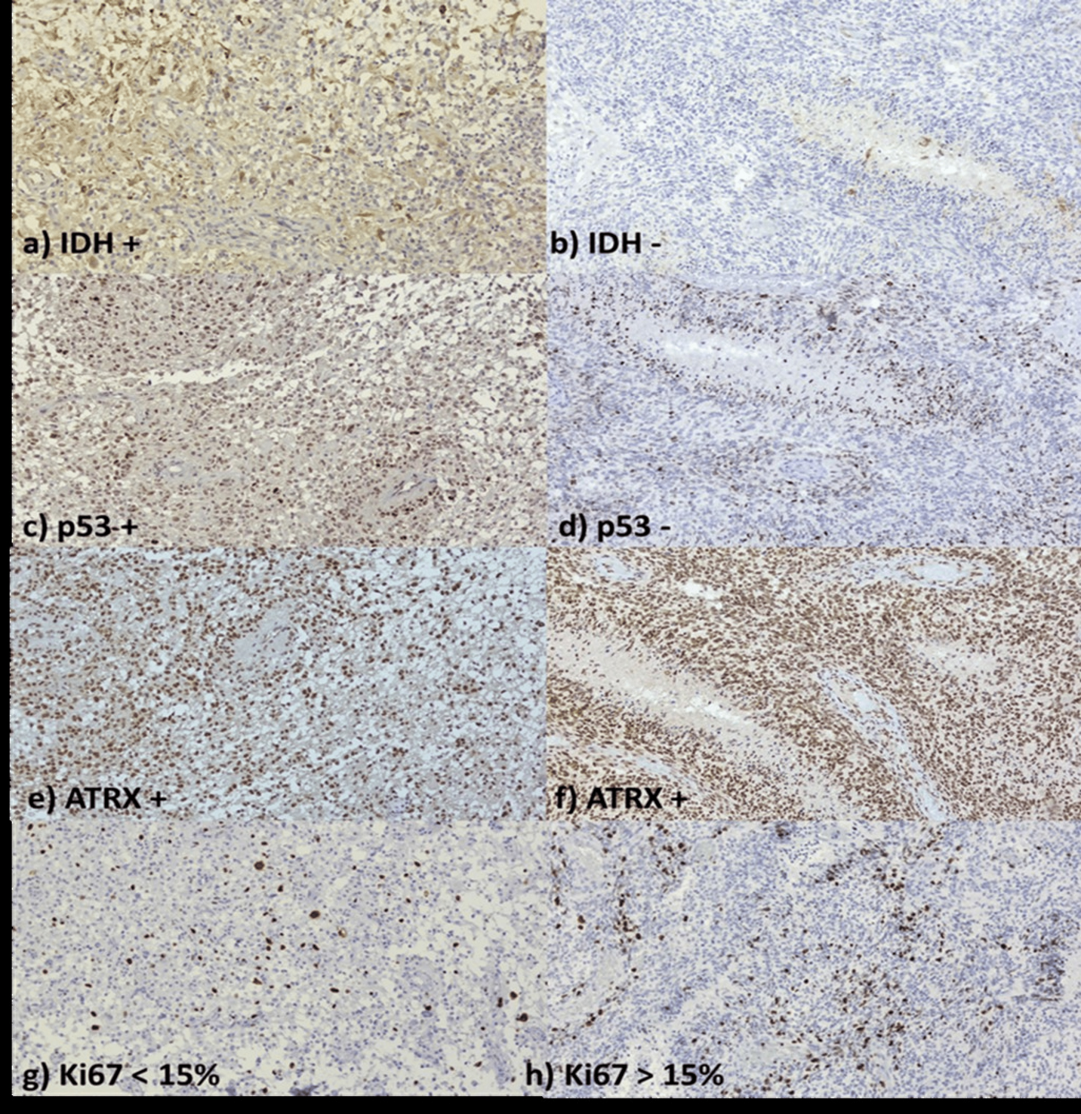
IHC Expression of Key Markers (IDH1-R132H, ATRX, p53, and Ki67) in the ASTROG4 and GBM a, c, e, g) Case No. 2, diagnosed with ASTROG4, OS: 26 months; b, d, f, h) Case No. 18, diagnosed with GBM, OS: 21 months. IHC analysis was performed on 4 μm paraffin-embedded sections using primary antibodies for IDH1-R132H, ATRX, p53, and PKM2. Detection was archived using the EnVision FLEX/HRP system and DAB chromogen. Positive expression criteria: IDH1-R132H >10% stained cells, ATRX loss <50% stained cells, p53 and Ki-67 based on nuclear staining in 200 and 1,000 cells, respectively. Resolution: 10x. IHC: immunohistochemistry, IDH: isocitrate dehydrogenase, ASTROG4: grade 4 astrocytoma, GBM: glioblastoma, OS: overall survival, PMK2: pyruvate kinase M2, HRP: horseradish peroxidase, DAB: diaminobenzidine

The evaluation of the IHC reactions was carried out independently by two qualified pathologists for the following reactions: IDH1, ATRX, p53, and Ki-67. Furthermore, to assess potential sources of interobserver bias, a standardized protocol was used for the evaluation of IHC reactions, and discrepancies between the two qualified pathologists were resolved through consultations and consensus. A preliminary examination of the sections was conducted using a Zeiss (Oberkochen, Germany) Axiolab 5 optical microscope equipped with an Axiocam 208 color digital camera. The Ki-67 proliferation index was determined as the percentage of tumor cells with positive nuclear staining (regardless of intensity) in 1,000 cells, while p53 evaluation was based on the percentage of cells showing nuclear immunostaining in 200 cells across five fields. Immunoexpression was considered negative if the immunostaining was <10% (wild-type) and positive if >10% of the examined cells were stained (mutant). IDH mutation expression was determined by assessing tumor cells with positive cytoplasmic immunostaining, regardless of intensity. Cases where at least 10% of cells were stained were considered positive (mutant IDH), while cases with staining in ≤10% of tumor cells were considered negative (wild-type IDH), as shown in Figure 1. For the ATRX marker, ATRX gene mutations result in the loss of nuclear immunoexpression in tumor cells, whereas ATRX immunoexpression remains preserved in non-mutated ATRX cells or endothelial cells, which also serve as an endogenous positive control. Cases with nuclear staining in more than 50% of cells were defined as ATRX-positive, while cases with <50% stained cells were defined as ATRX-negative (loss of ATRX expression).

Quantitative IHC

For the evaluation of IHC reactions for PKM2, the slides were digitally scanned at a 20x resolution using the Panoramic Digital scanner (3DHISTECH, Budapest, Hungary) and subsequently subjected to digital image analysis using the QuantCenter software (3DHISTECH). From each specimen, three areas of 1 mm2 each (resolution 20x), containing only tumor tissue, were identified, with each region containing over 700 tumor cells. To objectively evaluate IHC staining, we used the quantification module (CellQuant), which quantifies both the number and intensity of the immunostaining. Within the 3DHISTECH image analysis software, the H-score provides a more detailed assessment than simple positive cell counting, as it combines both the proportion of positive cells and their staining intensity, thereby quantifying variability in expression based on intensity.

Immunostaining was quantified using the CellQuant module from 3DHISTECH for digital image analysis. The quantification process involved selecting the “Quant Detection” option, which enabled color deconvolution to separate the specific signals of the chromogen in the plasma and the counterstain used to highlight the nuclei. Settings for the cell nuclei were adjusted with a minimum size of 6.0 and intensity at 1, while for the cytoplasm, the width was set to 3.0, with intensity at 1. Additionally, intensity for the membrane was set to 1. The analysis was performed on a region selected from the images, previously marked with Annotation1, 2, 3 using a 20x objective, which included tumor tissue. These settings allowed precise quantification of the intensity and the number of immunolabeled cells in each selected region.

**Figure 2:**
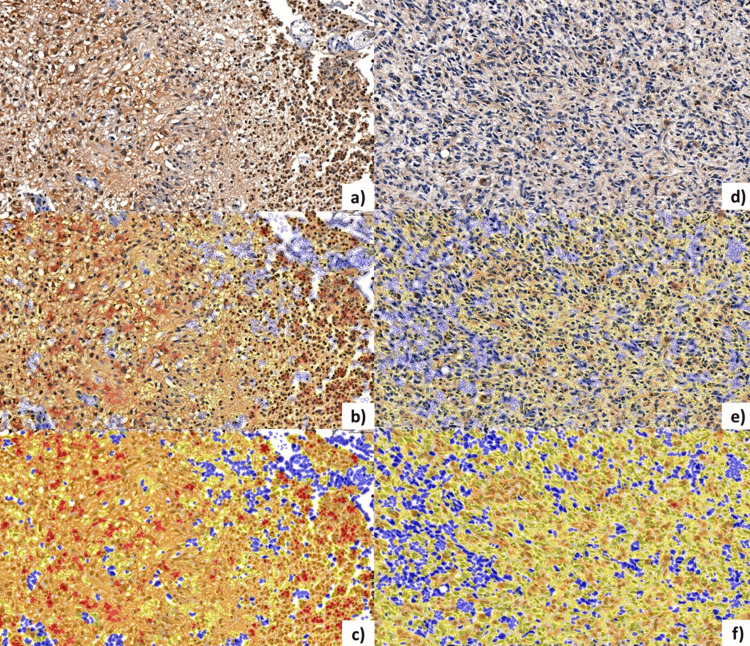
Immunohistochemical Quantification of PKM2 Expression in GBM and ASTROG4 Cohorts From the GBM cohort: a, b, c) Case No. 2: 50 years old, Female. Negative Index: 11.00%, H-score: 170.06, Intensity Score: 2, Proportion Score: 5, Resolution: 40x. From the ASTROG4 cohort: d, e, f) Case No. 8: 51 years old, Male. Negative Index: 22.65%, H-score: 102.27, Intensity Score: 1, Proportion Score: 5. Resolution: 40x. a, d) IHC marking with GFAP; b, e) Quantification via CellQuant "Outline mode"; c, f) Quantification via CellQuant "Fill mode" PMK2: pyruvate kinase M2, GBM: glioblastoma, ASTROG4: grade 4 astrocytoma, IHC: immunohistochemistry, GFAP: glial fibrillar acid protein

The H-score for each region was calculated using the default setting, which is determined by the percentage of positive cells and their intensity. The formula for H-score is: H-score = Σpi(i+1), where “pi” represents the percentage of positive cells out of the total number of cells, and “i” represents the intensity. The intensity score “i” can range from 0 to 3, where: 0- no staining, 1- weak staining, 2- moderate staining, 3- strong staining. The final H-score ranges from 0 to 300, where 0 indicates the absence of staining and 300 indicates staining present in 100% of cells. Regarding the interpretation of the H-score, a value between 0-100 suggests low or absent expression of the marker, a value between 100-200 indicates moderate expression, and a value between 200-300 suggests high expression of the marker. The Proportion Score quantifies the proportion of positive cells, representing the percentage of positive cells relative to the total cells in the tissue section. A proportion score of 0 indicates that 0% of cells are positive, a score of 1 indicates positivity between 1-10%, a score of 2 indicates positivity between 11-25%, a score of 3 indicates positivity between 26-50%, a score of 4 indicates positivity between 51-75%, while a score of 5 indicates positivity between 76-100% (Figure 2).

Statistics and receiver operating characteristic analysis

The statistical analysis included both descriptive statistics (mean, median, and standard deviation (SD)) and inferential statistics. For categorical variables, the chi-square test was used. For quantitative variables with a normal distribution, data was presented as mean and standard deviation, and the Student's t-test was used. For quantitative variables with a non-normal distribution (presented as median and range), the Mann-Whitney U test was employed. The Mantel-Cox test was used to obtain p-values, and Spearman correlation tests were conducted. The significance threshold for p was set at 0.05 with a 95% confidence interval. All statistical analysis was performed using GraphPad Prism 8 (GraphPad Software, San Diego, CA, USA) and IBM SPSS Statistics for Windows, version 27 (IBM Corp., Armonk, NY, USA).

## Results

Clinical and imaging characteristics

Between 2021 and 2023, a total of 144 patients were admitted to the Neurosurgery Department with clinical and imaging suspicion of high-grade glioma. Of these, only 67 (46.5%) cases met the study's inclusion criteria: 43 (48%) cases from the GBM group and 24 (44.4%) cases from the ASTROG4 group. The decision to perform histological evaluation on all cases (n=67) before dividing them into the GBM and ASTROG4 groups ensured a consistent and unbiased assessment. This methodology allowed us to apply uniform histological criteria across the cohort, which is critical for reliable comparisons.

The gender distribution was balanced in both groups, with an equal proportion of male and female patients in the ASTROG4 group (50%), while the GBM group showed a slight predominance of male patients (51.2%). Regarding patient age, there was a statistically significant difference between the two patient groups (p=0.001), with the average age at diagnosis being at least 10 years younger in the ASTROG4 group compared to the GBM group. In terms of the clinical presentation at admission, the main initial symptoms were minor motor deficit (46.5%) and aphasia (41.9%) in the GBM group, while aphasia (54.2%) and epileptic seizures (41.7%) were more common in the ASTROG4 group. The frontal lobe was most frequently involved in tumor development, affecting 46.5% of GBM cases and 56.5% of ASTROG4 cases (Table 1).

**Table 1:** Clinical and imaging characteristics of GBM versus ASTROG4 Statistical analyses were conducted using the Chi-square test (χ2) for categorical variables (e.g., sex, clinical presentation symptoms, tumor location, and laterality) and the Student's t-score for independent samples for continuous variables (e.g., age and tumor dimensions). A 95% confidence interval (CI) was assumed for all statistical tests, with significance set at p<0.05 ASTROG4: grade 4 astrocytoma, GBM: glioblastoma, IHC: intracranial hypertension

Clinical Characteristics: n(%)			GBM	ASTROG4	p-value	Chi-Square / t-Score
Sex: n(%)	Males		22 (51.2%)	12 (50%)	0.927	χ2 = 0.01
	Females		21 (48.8%)	12 (505%)	0.927	χ2 = 0.01
Age (years): mean ± standard deviation (SD)			60.4 ± 11.82	50.38 ± 10.63	0.001	t = 3.77
Clinical Presentation: n(%)	Headache		5 (11.6%)	2 (8.3%)	0.673	χ2 = 0.18
	ICH		11 (25.6%)	2 (8.3%)	0.087	χ2 = 2.94
	Minor motor deficit		20 (46.5%)	11 (45.8%)	0.957	χ2 = 0.00
	Major motor deficit		7 (16.3%)	4 (16.7%)	0.967	χ2 = 0.00
	Aphasia		18 (41.9%)	13 (54.2%)	0.333	χ2 = 0.94
	Epileptic seizures		13 (30.2%)	10 (41.7%)	0.345	χ2 = 0.89
	Mental status impairment		13 (30.2%)	5 (20.8%)	0.405	χ2 = 0.69
Imaging Characteristics: n(%)	Location	Frontal lobe	20 (46.5%)	13 (56.5%)	0.438	χ2 = 0.60
		Parietal lobe	11 (25.6%)	4 (17.4%)	0.449	χ2 = 0.57
		Temporal lobe	29 (32.6%)	12 (52.2%)	0.120	χ2 = 2.42
		Occipital lobe	11 (25.6%)	1 (4.3%)	0.033	χ2 = 4.58
		Insular lobe	2 (4.7%)	2 (8.7%)	0.512	χ2 = 0.43
	Laterality	Right	22 (51.2%)	11 (47.8%)	0.796	χ2 = 0.07
		Left	20 (46.5%)	12 (52.2%)	0.661	χ2 = 0.19
	Tumor sizes (mm): mean ± SD	Length	46.58 ± 16.07	48.19 ± 17.54	0.716	t = 0.36
		Width	42 ± 14.72	42.67 ± 11.69	0.857	t = 0.18
		Thickness	44.81 ± 12.31	45.57 ± 10.69	0.810	t = 0.24

IHC characteristics: quantitative analysis of PKM2

**Figure 3:**
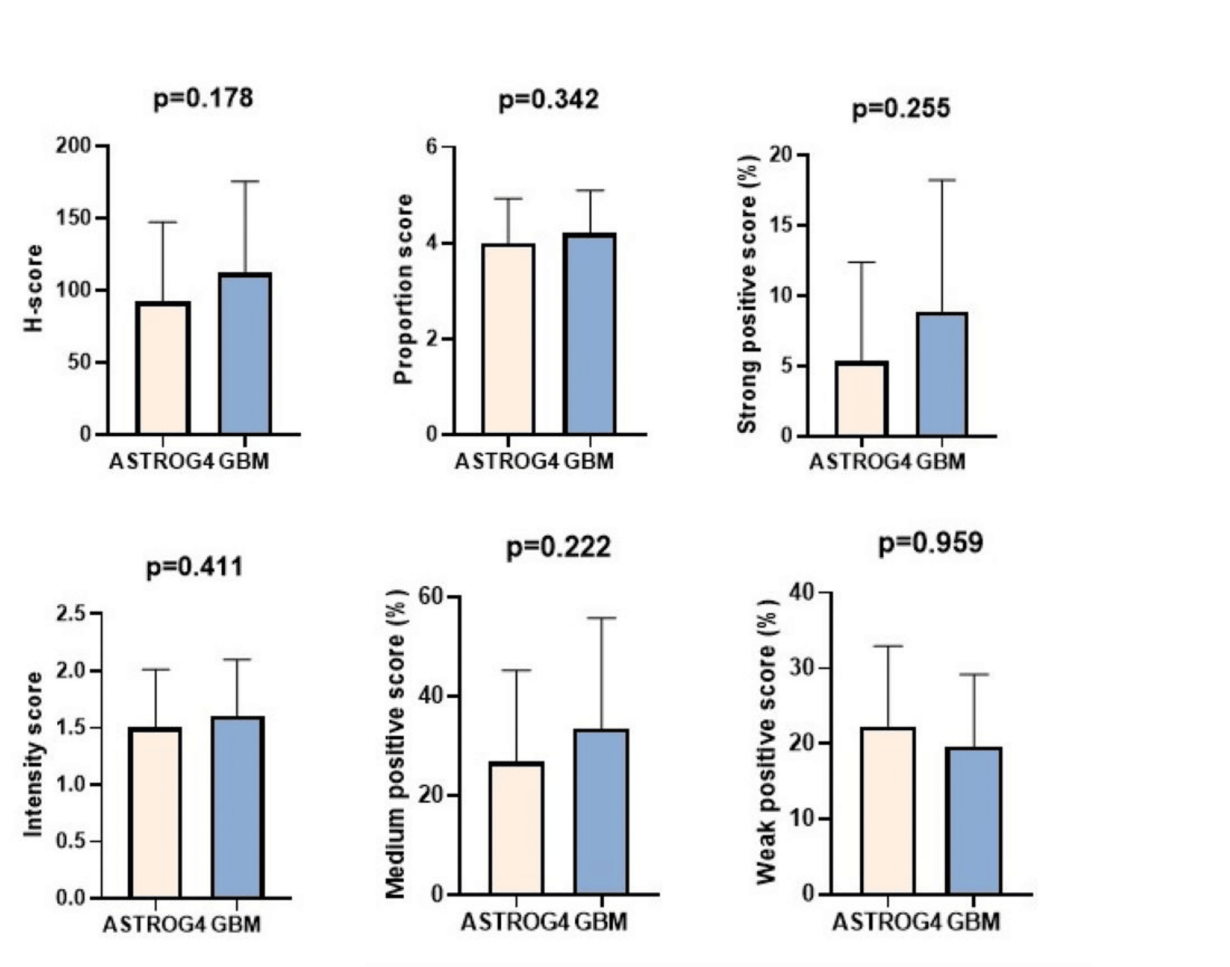
The comparative analysis of PKM2 expression between the ASTROG4 (N=24) and GBM (N=43) groups PKM2 – Negative (%): The mean ± SD for the ASTRGO4 group was 45.51 ± 26.47 and for the GBM group was 38.39 ± 28.09, showing no significant difference (p=0.691); PKM2 – Weak positive (%): The ASTROG4 group had a mean ± SD of 22.30 ± 10.62, while the GBM group had 19.64 ± 9.54, with no significant difference (p=0.959); PKM2 – Medium positive (%): The mean ± SD for ASTRGO4 was 26.83 ± 18.37 and for GBM was 33.53 ± 22.19, with no significant difference (p=0.222); PKM2 – Strong positive (%): The mean ± SD for ASTRGO4 was 5.34 ± 7.03 with a median of 2.41 [0.03-23.78], and for GBM it was 8.42 ± 8.95 with a median of 6.29 [0.00-30.68], with no significant difference (p=0.255); PKM2 – Intensity score: The mean ± SD for ASTROG4 was 1.5 ± 0.51 with a median of 1.5, and for GBM it was 1.6 ± 0.5 with a median of 2, showing no significant difference (p=0.411). PKM2 – Proportion score: The AS-TROG4 group had a mean ± SD of 4 ± 0.93 with a median of 4 (2-5), and the GBM group had a mean ± SD of 4.21 ± 0.88 with a median of 4 (2-5), with no significant difference (p=0.342). PKM2 – H-score: The mean ± SD for ASTROG4 was 92 ± 55.09 with a median of 92.5 [7.94-186.54], and for GBM it was 111.98 ± 63.25 with a median of 113.5 [4.84-223.97], showing no significant difference (p=0.178). Statistical analyses were performed using: Student's t-test for normally distributed continuous variables (mean ± SD); Mann-Whitney U test for non-normally distributed continuous variables (median and range). These results demonstrate that there were no statistically significant differences between the two groups for any of the PKM2-related parameters. ASTROG4: grade 4 astrocytoma, GBM: glioblastoma, PMK2: pyruvate kinase M2

All tissue samples from the patients included in the study showed positive glial fibrillar acid protein (GFAP) expression. All patients in the ASTROG4 group exhibited IHC expression of IDH1 R132H. p53 expression was observed in 83.33% (n=20) of ASTROG4 cases and in 53.48% (n=23) of GBM cases. Regarding ATRX expression, an inverse correlation with p53 was observed: positive ATRX expression was present in 86.04% of GBM cases and in 58.33% of ASTROG4 cases. The mean Ki67 proliferation index was 19.44% in the GBM group and 15.37% in the ASTROG4 group. For PKM2 expression, no statistically significant difference was found between the two groups (Figure 3).

In the GBM group, the PKM2 positivity rate was 61.6% distributed as follows: 19.64% weak positive, 33.53% medium positive, and 8.42% strong positive. In the ASTROG4 group, the positivity rate was 54.49%, with the following distribution: 22.3% weak positive, 26.83% medium positive, and 5.34% strong positive. Although the p-value was not statistically significant, a significant correlation was found between the mean positive expression (p=0.041), strong positive expression (p=0.004), intensity score (p=0.004), and H-score (p=0.019) of PKM2 and ATRX ICH expression in both patient groups (Table 2). The results presented in Table 2 represent the mean ± SD or medians, where applicable, for each analyzed parameter. The PKM2 scores were obtained through detailed IHC evaluation and digital analysis, as described in the Materials and Methods section. These evaluations were performed on all included cases (n=67), with histological confirmation prior to cohort differentiation into GBM and ASTROG4 groups.

**Table 2:** The comparison of PKM2 expression between different subgroups based on the presence of specific biomarkers, including IDH, ATRX, p53, and Ki67 ^1^For categorical variables, the results of the chi-square test are reported. For quantitative variables with a normal distribution (data presented as mean and SD), the Student's t-test was applied. For quantitative variables with a non-normal distribution, the Mann-Whitney U test was performed (95% CI) ^2^p-value < 0.01 ^3^Spearman correlation tests were applied For each subgroup, the table shows the mean ± SD of PKM2 expression as a percentage and the distribution of PKM2 staining intensity across four categories: Negative, Weak positive, Medium positive, and Strong positive. The intensity score, proportion score, and H-score are also presented, representing the strength of PKM2 staining, the percentage of positive cells, and a combined measure of both intensity and proportion of positive cells, respectively. Additionally, p-values for statistical compar-isons are included to assess the significance of differences between subgroups. For PKM2 versus ATRX, significant differences were found in the strong positive expression (p=0.0042) and H-score (p=0.019). However, no significant differences were observed for PKM2 versus IDH or PKM2 versus p53, with all p-values greater than 0.05. For PKM2 versus Ki67, correlation coefficients suggest a relationship, but none of the p-values reached statistical significance IDH: isocitrate dehydrogenase, ASTROG4: grade 4 astrocytoma, GBM: glioblastoma, PMK2: pyruvate kinase M2

	PKM2 (%)	Negative	Weak positive	Medium positive	Strong positive	Intensity score	Proportion score	H-score
PKM2 versus IDH: mean ± SD	IDH mutant (ASTROG4)	45.51± 26.47	22.30 ± 10.62	26.83 ± 18.37	5.34 ± 7.03	1.5 ± 0.51	4 ± 0.93	92 ± 55.09
	IDH wild type (GBM)	38.39 ± 28.09	19.64 ± 9.54	33.53 ± 22.19	8.42 ± 8.95	1.6 ± 0.5	4.21 ± 0.88	111.98 ± 63.25
	p-value^1^	0.691	0.959	0.222	0.255	0.411	0.342	0.178
PKM2 versus ATRX: mean ± SD	ATRX+	37.41 ± 26.5	19.58 ± 8.35	34.05 ± 20.38	8.94 ± 8.88	1.67 ± 0.47	4.22 ± 0.9	114.53 ± 60.35
	ATRX-	52.21 ± 28.58	23.82 ± 13.72	21.81 ± 20.89	2.15 ± 3.11	1.25 ± 0.447	3.88 ± 0.88	73.9 ± 52.74
	p-value^1^	0.06	0.258	0.041	0.004^2^	0.004	0.136	0.019
PKM2 versus p53: mean ± SD	p53+	40.77 ± 28.18	21.25 ± 10.87	31.19 ± 20.54	6.77 ± 8.11	1.56 ± 0.5	4.19 ± 0.93	103.97 ± 60.48
	p53-	41.25 ± 26.93	19.42 ± 8.11	31.02 ± 22.28	8.29 ± 8.97	1.58 ± 0.5	4.04 ± 0.86	106.36 ± 62.64
	p-value^1^	0.946	0.475	0.975	0.844	0.843	0.398	0.879
PKM2 versus Ki67: mean ± SD	Ki67 (%) Correlation Coefficient	-0.189	0.115	0.192	0.230	0.157	0.199	0.220
	p-value^3^	0.125	0.352	0.120	0.061	0.205	0.106	0.074

Survival and follow-up: clinical prognostic factors

**Figure 4:**
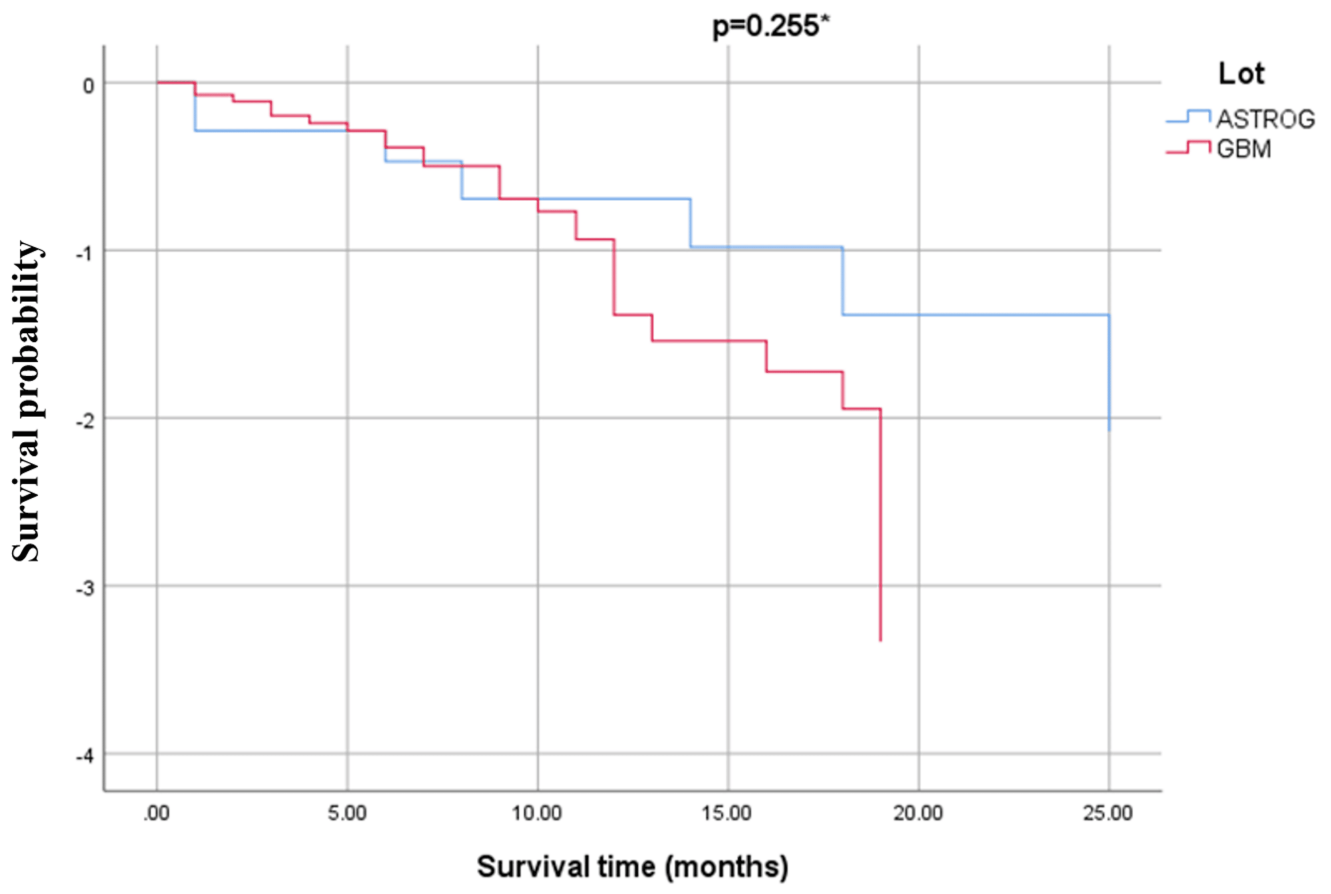
Kaplan-Meier survival curve for patients based on their group (GBM vs. ASTROG4) *p was obtained from the log-rank test (Mantel-Cox) A Kaplan-Meier survival curve comparing the cumulative survival probabilities (Y-axis) over time in months (X-axis) between ASTROG and GBM groups. The statistical comparison (p = 0.255) suggests no significant difference between groups ASTROG4: grade 4 astrocytoma, GBM: glioblastoma

The 12-month mortality rate was 39.5% (n=17) in the GBM group and 20.8% (n=5) in the ASTROG4 group, with a p-value of 0.118. The 24-month survival rate was 66.7% (n=16) for the ASTROG4 group and 25.6% (n=11) for the GBM group, with a p-value of 0.001. Figure 4 presents the Kaplan-Meier survival curve based on the patient groups and statistical data related to survival. The negative B coefficient suggests that ASTROG4 patients have a lower risk of death over the analyzed time period compared to the GBM group, although this was not statistically significant (p=0.275; p obtained using the log-rank test, Mantel-Cox). Additionally, based on the exponential value of the B coefficient, ASTROG4 patients have a 36.7% lower risk of death compared to the GBM patients.

Preoperative clinical factors and Cox regression analysis

**Figure 5:**
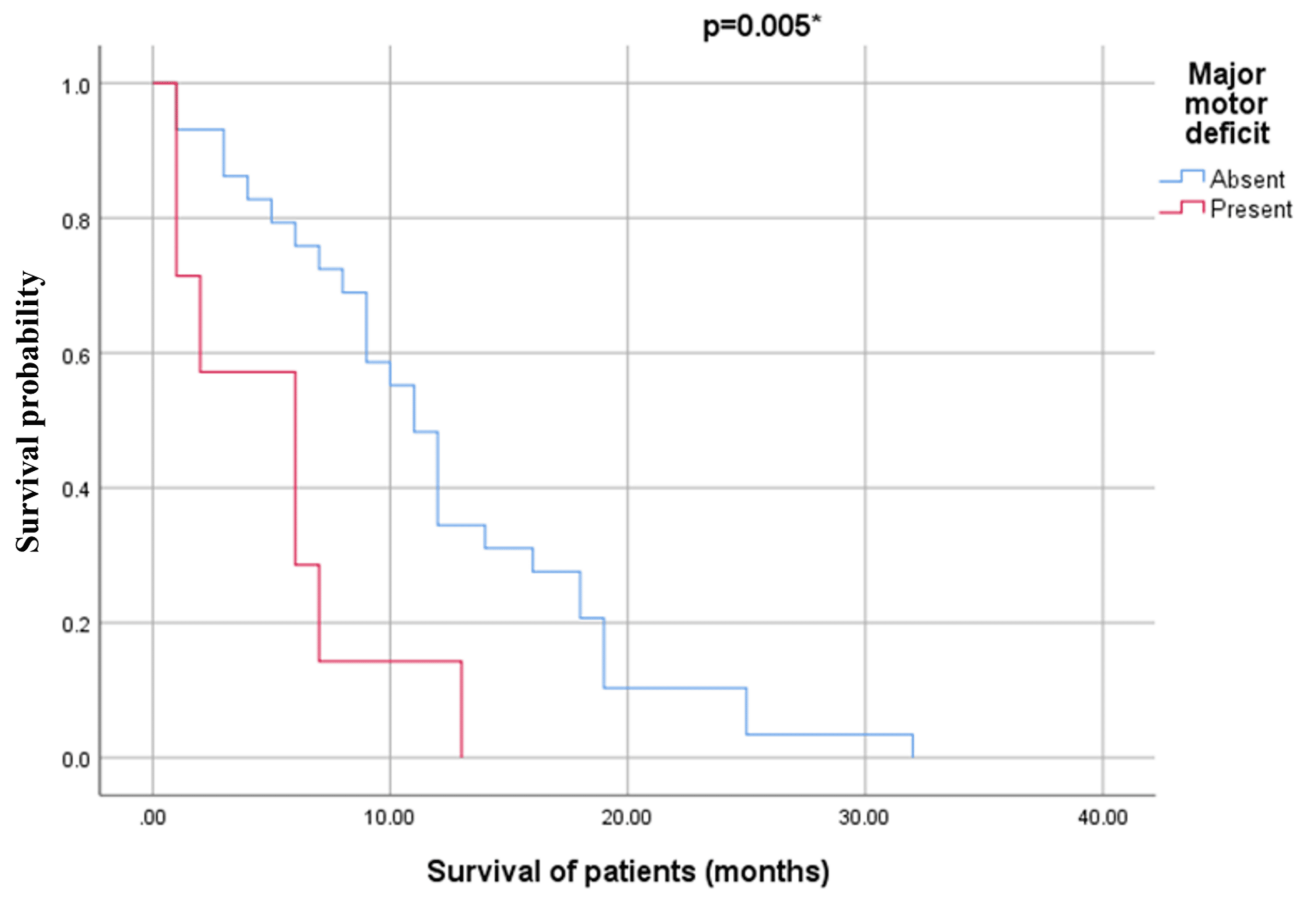
Kaplan-Meier survival curve of patients based on the presence of major motor deficit, regardless of group *p was obtained from the log-rank test (Mantel-Cox) The Y-axis represents the survival probability over time. As indicated by the results of our logistic regression analysis, the presence of a major preoperative motor deficit significantly affects the likelihood of poorer survival outcomes. The Wald statistic for this factor was 6.535 (p = 0.011), indicating that patients with major motor deficits have a significantly higher risk of death. The odds ratio (Exp(B) = 3.205) suggests that the likelihood of poor survival outcomes is approximately 3.2 times greater in patients with major motor deficits compared to those without

A statistically significant correlation was observed between the presence of major preoperative motor deficits and mortality. Cox regression analysis showed that patient survival is strongly influenced (p=0.02; p obtained using the log-rank test, Mantel-Cox), by the presence of major preoperative motor deficit. According to the results presented in Figure 5, the presence of a major motor deficit increases the probability of death by approximately 3.2 times. Major motor deficit is the only clinical parameter that determines survival in the patients included in our study.

Cox logistic regression analysis indicates that, overall, patient survival is not influenced by tumor type (ASTROG4 vs. GBM) in relation to PKM2 activity. However, among all the IHC parameters of PKM2 analyzed, the intensity score significantly influenced survival, with a p-value of 0.041. Each unit increase in the intensity score increases the probability of death by approximately 7.6 times (Table 3).

**Table 3:** Correlation between the quantitative IHC analysis of PKM2 and patient survival The overall adequacy of the model was evaluated using a chi-square test (Omnibus Test of Model Coefficients), which indicated no significant deviation from the data (Chi-square = 5.783, df = 6, p = 0.448). Significant associations were found for the PKM2-Intensity Score (B = 2.028, p = 0.041), with an odds ratio of 7.596, suggesting that higher intensity scores of PKM2 expression are associated with poorer survival outcomes IHC: immunohistochemistry, PMK2: pyruvate kinase M2

Variable	B	SE	Wald	df	Sig.	Exp(B)
Constant	-0.908	0.520	3.050	1	0.081	0.403
PKM2- Negative Score	-0.032	0.049	0.417	1	0.518	0.969
PKM2- Weak Positive	0.019	0.035	0.298	1	0.585	1.019
PKM2- Medium Positive	-0.020	0.043	0.207	1	0.649	0.980
PKM2- Intensity Score	2.028	0.991	4.190	1	0.041	7.596

## Discussion

The aim of this study was to assess the quantitative differences in PKM2 expression between GBM (IDH wild type) and ASTROG4 (IDH R132H mutant), as well as the impact of these differences on OS, to determine whether PKM2 could serve as a prognostic marker. Additionally, we investigated the relationship between PKM2 expression and commonly used IHC markers in astrocytic tumors, ATRX and p53, to evaluate their prognostic significance in the context of PKM2 expression.

Negative ATRX status in tumor cells, coupled with concurrent positive status in normal cells, suggests an ATRX mutation. Diffuse immunopositivity for p53, on the other hand, is indicative of a TP53 mutation. Loss of ATRX expression (negative status) due to mutations, deletions, gene fusions, or other molecular alterations leads to DNA damage. ATRX status has been associated with patient age and prognosis. ATRX mutations have been identified in children with pilocytic astrocytoma that occasionally exhibit signs of anaplasia. In adults, negative ATRX status has been observed in 71% of grade 2 and 3 astrocytoma, in up to 57% of grade 4 astrocytoma, and in only 4% of GBM cases. Negative ATRX status is thus more common in younger patients and is presumed to be a positive prognostic factor. Furthermore, ATRX mutations are specific to astrocytoma and help differentiate them from oligodendrogliomas, where 1p19q codeletion rarely co-occurs with ATRX mutations. Another critical aspect is the involvement of ATRX loss (negative status) in immune response, potentially increasing cytokine secretion, inflammatory infiltrates, and even tumor cell growth inhibition by immune cell effectors [27,7].

Our findings support these observations. Negative ATRX status was found in only 13.96% of the GBM lot and in 41.67% of the ASTROG4 lot, aligning with the distinct genetic alteration patterns characteristic of IDH-mutant astrocytoma. Similarly, p53 expression was more frequent in the ASTROG4 group (83.33%) compared to the GBM group (53.48%). These differences confirm the correlation between IDH mutations and more stable tumor progression. Our results align with literature findings suggesting that p53 expression is frequently associated with IDH mutations (Table 2). According to the literature, the combined IDH/p53 mutation is characteristic of astrocytoma, implying more stable tumor evolution and greater sensitivity to specific therapies. In GBM, p53 is a less specific marker, but is thought to be associated with more aggressive progression and should be interpreted alongside other molecular biomarkers for a more comprehensive profile [28-30].

PKM2 overexpression has been noted in various cancer types. Park et al. provided detailed insights into the IHC expression of PKM2 in glioma tissue samples, adjacent non-tumorous tissues, and normal brain tissue. Their findings demonstrated that PKM2 overexpression is characteristic of tumor tissue and is correlated with glioma development. PKM2, a key regulator of cellular energy metabolism, is significantly overexpressed in tumor tissues compared to normal brain tissues. This suggests its significant role in the initiation and progression of brain tumors. Notably, the study also reported increased PKM2 expression in normal tissues adjacent to tumors, though less pronounced than in glioma tissues. This finding hints at the possibility of genetic alterations occurring in the peripheral regions surrounding the tumor [20].

In our study, we employed digital quantitative measurement for slide visualization and analysis to minimize interobserver variability. The analyzed area included over 700 cells/mm2. Digital analysis provides both a binary assessment (positive or negative) of PKM2 expression and detailed scoring evaluations. This approach allowed for precise and reproducible measurements of PKM2 expression, which is crucial in understanding its potential role in tumor metabolism. Comparing PKM2 expression with IDH mutation status revealed no significant differences between IDH-mutant (ASTROG4) and IDH-wild type (GBM) cases. Intensity scores, proportion scores, and H-scores were similar across both groups, with no statistically significant differences (p > 0.05, Table 2, Figure 3). These findings suggest that PKM2 expression is independent of IDH status in high-grade astrocytic tumors. Regardless of whether a tumor is IDH-mutant or IDH-wild type, PKM2 expression remains consistent, indicating that PKM2 may not be directly influenced by the IDH mutation in the context of GBM and ASTROG4. Thus, PKM2’s role in tumor metabolism and progression may involve distinct molecular pathways, such as those related to glycolysis or alternative metabolic reprogramming mechanisms, warranting further investigation into its potential as a therapeutic target in both IDH-mutant and IDH-wild type tumors.

On the other hand, when examining the relationship between PKM2 and ATRX expression, significant differences were observed. ATRX-negative cases exhibited higher PKM2 expression compared to ATRX-positive cases. This difference was statistically significant for medium-positive (p = 0.041) and intensely positive PKM2 expression (p = 0.004), as well as for calculated scores (p = 0.004 for intensity score and p = 0.019 for H-score, Table 2). This observation raises the hypothesis that ATRX and PKM2 are interconnected through cellular regulatory mechanisms. ATRX is a key marker for genomic stability. Its loss may increase energy demands in tumor cells, driving greater PKM2 activity in glycolytic processes to meet metabolic needs. Although ATRX itself is not directly involved in glycolysis, its role in maintaining chromatin stability and preventing telomere dysfunction is crucial for preserving cellular integrity. Loss of ATRX function may lead to metabolic shifts, favoring alternative pathways such as aerobic glycolysis to support tumor growth and invasiveness. These findings suggest that, in the absence of ATRX, tumor cells may develop an increased dependence on PKM2 activity. This relationship underscores their significance in oncogenesis and identifies new avenues for research into the metabolic mechanisms underlying glioma genesis. Further investigation into the ATRX-PKM2 axis could reveal novel therapeutic strategies targeting tumor metabolism.

We also examined the relationship between PKM2 and p53, finding no statistically significant correlation between these two markers. Regardless of p53 status (positive or negative), PKM2 expression levels remained similar, with no significant statistical differences (p > 0.05 for all categories). This result suggests that PKM2 activity is not influenced by p53 in high-grade astrocytoma, even though p53 is recognized as a major regulator of the cell cycle and apoptosis in this pathology. It is possible that PKM2 operates via pathways independent of those controlled by p53, potentially involving alternative cellular survival mechanisms. Additionally, correlating PKM2 with the proliferation marker Ki67 yielded statistically insignificant results. Although PKM2 is frequently associated with increased metabolic activity in tumors, its expression levels were not closely correlated with the cellular proliferation rate marked by Ki67 (p > 0.05, with a correlation coefficient of r = 0.230 for strong expression), as shown in Table 2. This finding suggests that PKM2 may play a more critical role in the metabolic adaptation of tumor cells rather than directly driving their growth. PKM2 might support tumor development by fulfilling energy demands rather than by directly stimulating cellular division. This metabolic-focused function makes PKM2 a unique therapeutic target for inhibiting tumor metabolism without affecting the proliferation of normal cells. The weak relationship between PKM2 and Ki67 supports the notion that PKM2 predominantly plays a metabolic role rather than a proliferative one. This distinction could inform therapeutic strategies targeting PKM2 to disrupt tumor metabolism selectively while sparing normal tissue functions.

Comparing survival outcomes between GBM and ASTROG4 is essential for understanding the prognostic differences between these two types of malignant brain tumors. Mortality within 12 months of diagnosis shows similar trend in both groups. A higher proportion of GBM patients (39.5%) succumbed within this period compared to ASTROG4 patients (20.8%). However, this difference is not statistically significant (p = 0.118). Both groups face a substantial risk of death within the first year, but this difference lacks statistical confirmation. In contrast, survival beyond 24 months reveals a statistically significant difference (p = 0.001). Approximately two-thirds (66.7%) of ASTROG4 patients survived more than 24 months, compared to only 25.6% of GBM patients. Further analysis using the coefficient B (negative), indicates that ASTROG4 patients have a 36.7% lower risk of death than GBM patients. However, this finding does not achieve statistical significance (p = 0.275), highlighting the need for cautious interpretation (Figure 4). These findings reinforce the well-documented notion that GBM is a significantly more aggressive tumor with a much poorer prognosis compared to ASTROG4. Differentiating these tumor types is crucial for discussing patient prognosis and tailoring treatment strategies effectively.

Cox regression analysis of survival data reveals a statistically significant effect of the PKM2 intensity score on patient survival (p = 0.041). The coefficient B = 2.028 indicates a positive association between the intensity score and mortality risk. For each unit increase in the PKM2 intensity score, the risk of death increases approximately 7.6 times (Exp(B) = 7.596, Table 3). This finding suggests that higher PKM2 intensity scores, indicative of elevated metabolic activity, are associated with more aggressive tumor behavior and faster disease progression. As the original aim of this study was to assess the differences in PKM2 expression between GBM (IDH wild type) and ASTROG4 (IDH R132H mutant), our findings suggest that PKM2 expression, particularly the intensity score, could serve as a prognostic marker of disease severity across both groups. Moreover, while no significant differences were found in PKM2 expression based on IDH mutation status, the intensity score might still provide valuable insights for patient stratification and personalized treatment planning.

An independent predictor of survival was the presence of significant motor deficit (MRC ≤ 3). The Stepwise Cox Regression analysis identified motor deficit as a statistically significant factor, with a coefficient B = 1.165 (p = 0.011). Patients with significant motor deficits had a hazard ratio (Exp(B) = 3.205), meaning they were approximately 3.2 times more likely to die within a given period compared to those without such deficits. This underscores the substantial impact of motor deficits on survival outcomes.

The global significance of the regression model was confirmed by the Omnibus Tests of Model Coefficients (Figure 5), which yielded a Chi-square value of 7.248 and a p-value of 0.007. Furthermore, the Stepwise Cox Regression analysis (p = 0.020) demonstrated that including motor deficits as a variable significantly improved the model's explanatory power for survival variability. These findings emphasize the prognostic importance of both the PKM2 intensity score and motor deficits in GBM and ASTROG4 patients. The PKM2 intensity score reflects tumor aggressiveness, supporting its use in risk stratification. Meanwhile, motor deficits emerge as a robust predictor of poor survival, highlighting the need for early intervention and tailored rehabilitation to improve patient quality of life and potentially enhance survival outcomes. Together, these insights offer valuable guidance for clinical decision-making and risk management in glioma care.

Although we have uncovered important data regarding the role of PKM2 in high-grade astrocytic tumors, this study has some notable limitations. The classification of high-grade astrocytic tumors was based exclusively on histopathological and IHC criteria, and the absence of genetic analyses could limit the overall accuracy of the findings. Genetic analyses would have provided a more comprehensive understanding of the tumor's molecular landscape, enhancing the interpretation of PKM2 as a potential prognostic marker. We acknowledge that this limitation may affect the reproducibility and generalizability of the study, as genetic mutations or alterations could influence PKM2 expression and tumor behavior in ways that were not captured by the current methodology. Furthermore, the histopathological evaluations were validated solely by our internal research team, which may have introduced a degree of subjectivity. We have made efforts to mitigate this potential bias by ensuring rigorous internal quality control measures. However, future studies would benefit from validation by an external team to further reduce any subjectivity. In addition, the study relied solely on IHC techniques to investigate the role of PKM2. While IHC is a valuable tool, incorporating other methods such as genomic or proteomic analyses could have provided more robust and complementary data, leading to a better understanding of PKM2's role in tumor progression. The relatively small cohort size is another limitation that could impact the statistical power of the study. Future research should include larger sample sizes to validate our findings and improve the reliability of the results.

To address these limitations, we implemented strategies to ensure the consistency of IHC staining. These included rigorous standardization of protocols and careful validation of antibody specificity, as well as the use of automated digital image analysis tools to reduce inter-observer variability. Furthermore, we made efforts to minimize selection bias during patient recruitment by employing clear inclusion and exclusion criteria. These strategies were crucial in enhancing the transparency of our methodology and the robustness of our findings. We recognize that these limitations highlight areas for improvement in future studies. Expanding the scope of research to include genetic profiling, larger sample sizes, and multi-modal analytical approaches would provide a more holistic understanding of PKM2's potential as a prognostic marker. Additionally, exploring strategies to further minimize bias and enhance reproducibility would strengthen the overall reliability of the results.

## Conclusions

Elevated PKM2 levels in ATRX-negative cases suggest a compensatory response to genomic instability associated with ATRX loss, while higher PKM2 intensity scores are linked to an increased risk of mortality. Moreover, PKM2 expression appears to be independent of IDH and p53 status, highlighting its distinct role in tumor progression. Additionally, differences in p53 expression between GBM and ASTROG4 highlight a more stable tumor evolution in ASTROG4.

Severe preoperative motor deficits (MRC ≤ 3) significantly increase the risk of death, emphasizing the importance of clinical factors in preoperative planning. By integrating clinical parameters with molecular biomarker analysis, we can refine prognosis and improve patient management. In conclusion, our findings highlight the importance of PKM2 and ATRX in high-grade glioma progression and suggest new directions for therapeutic strategies targeting the metabolic pathways involved in this process.
